# Non-invasive prenatal test identifies circulating cell-free DNA chromosomal abnormalities derived from clonal hematopoiesis in aggressive hematological malignancies

**DOI:** 10.1007/s10238-024-01313-3

**Published:** 2024-04-05

**Authors:** Valentina Giudice, Monica Ianniello, Danilo De Novellis, Luca Pezzullo, Nadia Petrillo, Bianca Serio, Matteo D’Addona, Anna Maria Della Corte, Michela Rizzo, Bianca Cuffa, Maria Antonietta Castaldi, Pasquale Savarese, Alessio Mori, Rosa Castiello, Antonio Fico, Giovanni Savarese, Carmine Selleri

**Affiliations:** 1 Hematology and Transplant Center, University Hospital “San Giovanni di Dio e Ruggi d’Aragona”, Salerno, Italy; 2 Department of Medicine, Surgery and Dentistry “Scuola Medica Salernitana”, University of Salerno, 84081 Baronissi, Italy; 3 Ames Center s.r.l, Casalnuovo di Naples, Naples, Italy; 4 Gynecology and Obstetrics Unit, University Hospital “San Giovanni di Dio e Ruggi d’Aragona”, Salerno, Italy

**Keywords:** Non-invasive prenatal test, Hematological malignancies, Screening, Prognosis, Diagnosis, Biomarker

## Abstract

Liquid biopsy is a minimally invasive diagnostic tool for identification of tumor-related mutations in circulating cell-free DNA (cfDNA). The aim of this study was to investigate feasibility, sensitivity, and specificity of non-invasive prenatal test (NIPT) for identification of chromosomal abnormalities in cfDNA from a total of 77 consecutive patients with non-Hodgkin B-cell lymphomas, Hodgkin lymphoma (HL), or plasma cell dyscrasia. In this case series, half of patients had at least one alteration, more frequently in chromosome 6 (23.1%), chromosome 9 (20.5%), and chromosomes 3 and 18 (16.7%), with losses of chromosome 6 and gains of chromosome 7 negatively impacting on overall survival (OS), with a 5-year OS of 26.9% and a median OS of 14.6 months, respectively (*P* = 0.0009 and *P* = 0.0004). Moreover, B-cell lymphomas had the highest NIPT positivity, especially those with aggressive lymphomas, while patients with plasma cell dyscrasia with extramedullary disease had a higher NIPT positivity compared to conventional cytogenetics analysis and a worse outcome. Therefore, we proposed a NIPT-based liquid biopsy a complementary minimally invasive tool for chromosomal abnormality detection in hematological malignancies. However, prospective studies on larger cohorts are needed to validate clinical utility of NIPT-based liquid biopsy in routinely clinical practice.

## Introduction

Molecular and genetic profiling of hematological malignancies is critical for disease management, risk stratification, and therapeutic decisions, and is currently performed on invasive biopsy specimens, such as bone marrow (BM), lymphnodes, or other involved tissues. However, this approach has limited diagnostic sensitivity and specificity, because bulky samples are mostly processed in routine clinical practice thus lacking to identify small subclones in complex specimens and to completely characterize tumor genomic and phenotypic heterogeneity [1].

Liquid biopsy is a minimally invasive procedure that allows detection of various circulating tumor-associated components, such as cell-free nucleic acids or exosomes [2, 3], for somatic mutation screening in cancer-associated genes and monitoring of minimal residual disease (MRD) [4, 5]. Circulating cell-free DNA (cfDNA) is shed by apoptotic or necrotic cells, such as highly proliferating cancer cells, and released into the bloodstream, thus being extremely representative of tumor tissue genomic heterogeneity, such as during Hodgkin (HL) and non-Hodgkin B-cell lymphomas [6–8]. Moreover, somatic variants detected in cfDNA can derive from clonal hematopoiesis, a branched evolution hematopoietic model in which multiple co-existing clones diverge and evolve in parallel with the acquisition of additional somatic mutations that might lead to tumor development [9]. Recurrent somatic mutations are found in most hematological malignancies, such as myelodysplasia, with disease-specific mutated gene patterns, have diagnostic and/or prognostic significance, and can be used as a pharmacological targets; however, clonal hematopoiesis is common in older general population with unknown clinical significance [10].

Current methods for liquid biopsy have several limitations, including the need of highly sensitive assays for small clone detection, discordant congruence between whole tissue and cfDNA assays with different genetic alterations, and not standarded data normalization [11–13], such as in myelodysplastic syndromes with low allele burdens or in healthy individuals with clonal hematopoiesis [14]. Non-invasive prenatal testing (NIPT) is a novel approach for detection of fetal cfDNA in maternal plasma during pregnancy and is used for non-invasive prenatal screening of fetal chromosomal abnormalities [15]. NIPT-based liquid biopsy has also proven to be a very sensitive and specific approach for chromosomal abnormalities derived from occult maternal primitive tumors [16, 17]. Indeed, during pregnancy, hormonal changes allow immune tolerance against the fetus (an allogeneic organism), thus reducing the alert status of the immune system also against cancer cells [17].

To investigate clinical utility of NIPT-based liquid biopsy for chromosomal abnormality detection in hematologic malignancies, a cohort of patients with multiple myeloma (MM), HL and B-cell lymphomas, and a small cohort of healthy donors were screened for cfDNA chromosomal alterations, and results were correlated with outcomes, including survival and response to treatment.

## Patients and methods

### Study cohort

A total of 78 patients diagnosed with hematological malignancies (plasma cell dyscrasias, *N* = 20; chronic lymphocytic leukemia [CLL], *N* = 22; nodular sclerosis HL [NSHL], *N* = 9 and lymphocyte depleted (LDCHL), *N* = 1; and B-cell lymphomas, *N* = 26) and healthy controls (*N* = 3), from 2016 to September 2023 at the Hematology and Transplant Center, University Hospital “San Giovanni di Dio e Ruggi D’Aragona”, Salerno, Italy, were included in this study. Patients received diagnosis and chemotherapy according to current international guidelines [18–20]. Inclusion criteria were: age ≥ 18 years old; new diagnosis of MM, CLL, HL, or B-cell lymphomas before treatment initiation; refractory/relapsed MM, CLL, HL, or B-cell lymphomas; and obtained written informed consent, in accordance with the Declaration of Helsinki and protocols approved by local Ethic Committee ‘‘Campania Sud’’ (Brusciano, Naples, Italy; prot./SCCE n. 24,988). Response criteria for plasma cell dyscrasias were the International Myeloma Working Group consensus criteria for response [21], and the International Working Group consensus response evaluation criteria in lymphoma (RECIL 2017) for B-cell lymphomas [22].

### Non-invasive pre-natal testing

Non-Invasive Prenatal Testing (NIPT) is a pre-natal screening carried out on maternal blood for a safe and accurate detection of chromosomal fetal abnormalities without performing invasive procedures, such as amniocentesis. Briefly, 10 mL of whole peripheral blood samples from hematological patients at diagnosis or at disease relapse were collected in special tubes for preventing clotting and ensure high DNA stability (STRECK Cell-Free DNA BCT, Streck Corporate, NE 68128, USA). Samples were kept and transported at 4 °C until processing within maximum 7 days from collection in an AMES laboratory, accredited (UNI EN ISO 9001:2008) for pre-natal and genetic disease testing. Here, specimens were processed according to manufacturers’ instructions of VeriSeq NIPT Solution v2 package insert. First, a first centrifugation at 1600 rcf, 4 °C for 10 min was performed, and subsequently 900 μL of supernatant consisting of separated plasma were transferred to a deep-well plate for an additional centrifugation at 5600 rcf, 4 °C for 10 min and used for cfDNA extraction. To enrich cfDNA of non-hematopoietic stem cell origin, magnetic beads and bioinformatic size-base separation techniques were employed. cfDNA extraction and purification were then achieved by adsorption onto a binding plate, washing to remove contaminants, and then by elution, as previously described [23]. The pipeline included an automated library preparation (VeriSeq NIPT Solution v2, Microlab STAR, assay) with subsequent whole genome sequencing (WGS) on NextSeq 550Dx, (Illumina Inc., San Diego, CA, USA). NIPT was performed using Illumina Next-Generation Sequencing (NGS) technology, for massively parallel sequencing of entire genomes with different resolving powers in all technical and analytical phases.

### Biostatistics pipeline

VeriSeq NIPT Assay Software version 2 (www.illumina.com/NIPTsoftware) was employed for analysis of the aneuploid status (detection of aneuploidy, hyperploidy, sex chromosomes and SCAs, rare autosomal aneuploidies or RAAs, and partial deletions/duplications ≥ 7 Mb, or CNVs) and for ctDNA levels (TF, Tumor Fraction) estimation. For the following bioinformatic analysis, generated WGS data were streamed to the VeriSeq NIPT Analysis Server (https://emea.support.illumina.com/content/dam/illumina-support/documents/documentation/chemistry_documentation/veriseq-nipt-v2/veriseq-nipt-solution-v2-software-guide-ivd-1000000067940-06.pdf, accessed on 1 December 2022). After data filtration and alignment to a human reference genome, a counting‐based algorithm was used to generate the log-likelihood ratio (LLR) scores for all chromosomes as well as NCV_X and NCV_Y scores for sex classification for each sample. LLR thresholds for calling a sample high or low risk were internally validated. Samples failed when sequencing coverage was judged insufficient based on TF, internal quality control parameters, and dynamic cut-offs of the VeriSeqTM NIPT Solution v2. When samples repeatedly failed due to data outside of expected range (DOER), a genome-wide data analysis was assessed with an in-house developed algorithm, as previously described [23]. Results were classified using the VeriSeq NIPT Solution v2 Assay Software NIPT (Illumina, Inc. 2022. VeriSeq NIPT Solution v2 Package Insert. 2021. Available online: https://support.illumina.com/downloads/veriseq-nipt-solution-v2-package-insert1000000078751.html; accessed on 1 December 2022), as previously reported [23, 24].

### Statistical analysis

Data were collected in spreadsheets and were analyzed using R statistical software (v. 4.0.5; RStudio) and SPSS (v. 25; IBM). VeriSeqtm NIPT Solution Assay Software v2 was used for common trisomies (13, 18, and 21) and sex chromosomes (SCA, Sexual Chromosome Aneuploidies), as well as for rare autosomal aneuploidies (RAAs), and partial deletions/duplications ≥ 7 Mb (copy number variation, CNVs) [25]. Categorical variables were expressed as counts and percentages and compared using Chi-square or Fisher’s exact test as appropriate. Log-rank (Mantel-Cox) test was used for survival analysis between groups. A *p* value of < 0.05 was considered statistically significant.

## Results

### Recurrent chromosomal abnormalities in hematological malignancies identified by NIPT

**Fig. 1:**
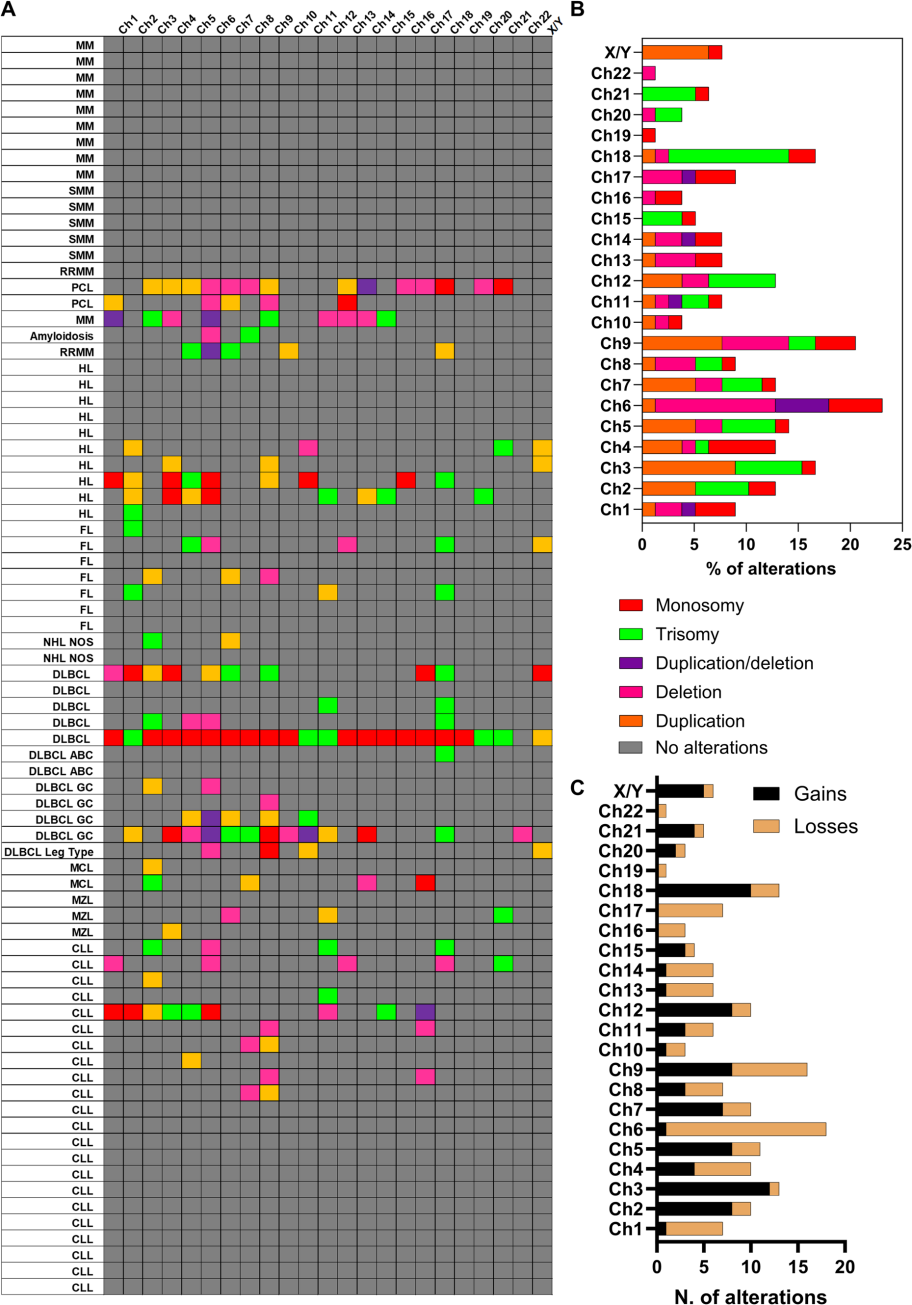
Chromosomal abnormality landscape and frequency distribution. (**A**) Landscape of chromosomal alteration distribution among study cohort (*N* = 78). Frequency distribution of alterations detected by NIPT by (**B**) type and (**C**) by category (gains or losses). *Abbreviations Ch* chromosome; *MM* multiple myeloma; *SMM* smoldering MM; *RRMM* refractory/relapse MM; *PCL* plasma cell leukemia; HL, Hodgkin lymphoma; FL, follicular lymphoma; NOS, not otherwise specified; *DLBCL* diffuse large B-cell lymphoma; *ABC* activated B-cell; *GC* germinal center; *MCL* mantle cell lymphoma; *MZL* marginal zone lymphoma; *CLL* chronic lymphocytic leukemia

To investigate the ability of NIPT-based liquid biopsy in identifying chromosomal abnormalities in cfDNA in various hematological malignancies, a total of 78 patients were screened at diagnosis or at disease relapse for chromosomal alterations (Fig. 1A). Thirty-nine subjects (50%) did not show any abnormalities by NIPT, while the remaining half of patients (*N* = 39) had at least one alteration (median, 3; range, 1–22), more frequently in chromosome 6 (*N* = 18; 23.1%), chromosome 9 (*N* = 16; 20.5%), chromosomes 3 and 18 (both *N* = 13; 16.7%), chromosome 5 (*N* = 11; 14.1%), and chromosomes 2, 4, 7, and 12 (*N* = 10 each; 12.8% each) (Fig. 1B). In particular, chromosome 6 abnormalities were predominantly deletions (*N* = 9; 11.5%), duplication of the short arm with deletion of the long arm (*N* = 4; 5.1%), or monosomy 6 (*N* = 4; 5.1%); conversely, gains of regions or chromosomes were more frequently observed in chromosomes 2 (10.3% vs. 2.6%, gains vs losses), 3 (15.4% vs. 1.3%), 7 (8.97% vs. 3.9%), 12 (10.3% vs. 2.6%), and 18 (12.8% vs. 3.9%) (Fig. 1C). Similar frequencies of gains and losses were described for chromosome 9 (*N* = 8 each; 10.2% each) and for chromosome 4 (*N* = 4 and *N* = 6, 5.1% and 7.7%, gains and losses, respectively).

**Fig. 2:**
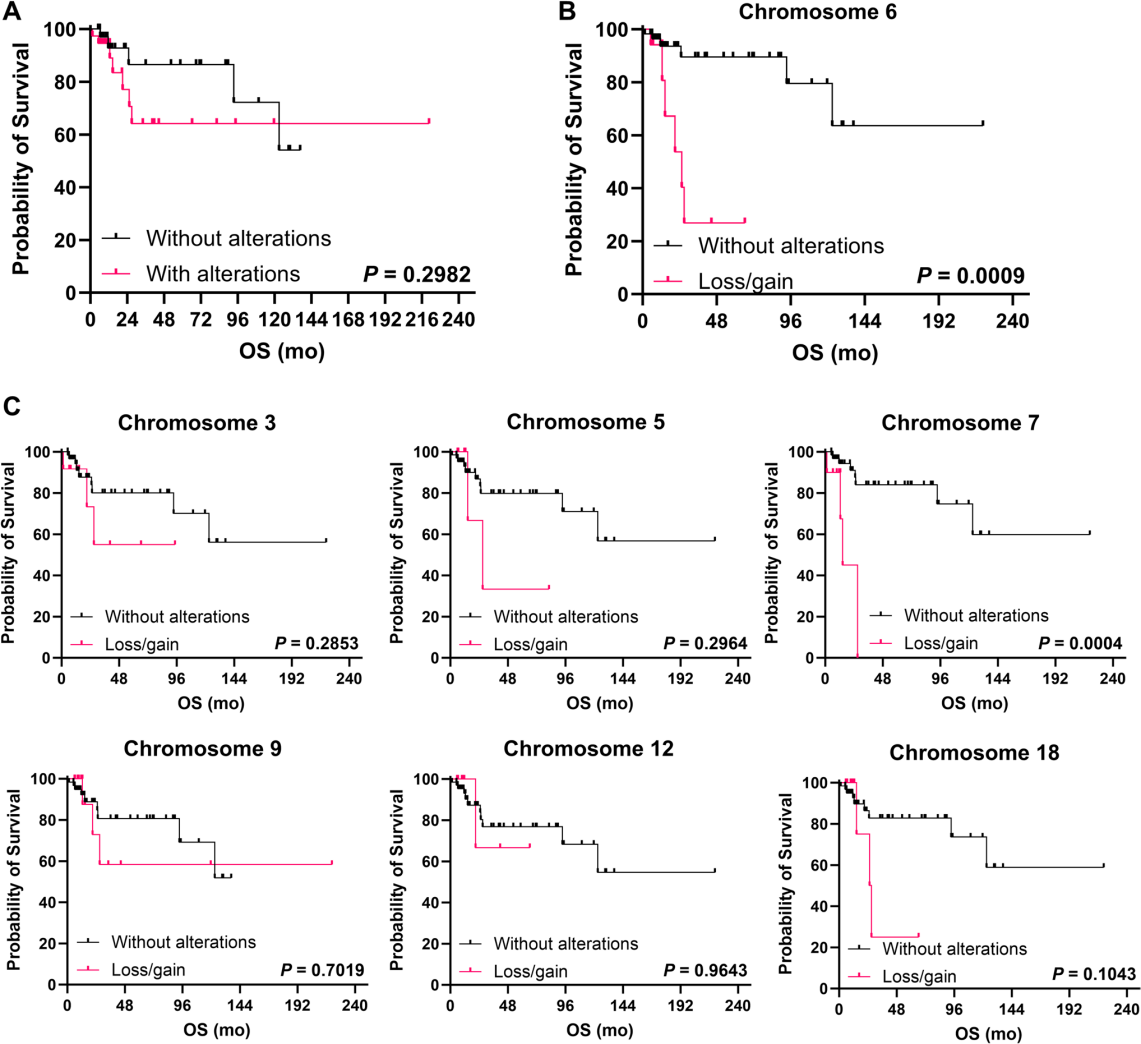
Clinical outcomes. Kaplan–Meier curves for overall survival (OS) of patients (**A**) with and without chromosomal abnormalities by NIPT, (**B**) with and without chromosome 6 abnormalities, and (**C**) with and without of chromosomes 3, 5, 7, 9, 12, and 18 alterations

Next, to study clinical impact of chromosomal abnormalities detected by NIPT in cfDNA, patients were divided based on absence or presence of any chromosomal alteration, and overall survival (OS) was compared (Fig. 2A). Despite no significant differences were observed between groups (*P* = 0.2982), 5-year OS of subjects with chromosomal abnormalities by NIPT tended to be shorter compared to those patients without alterations (64.2% vs. 86.6%). When divided based on specific chromosomal alterations, patients with gains or losses of chromosome 6 showed a significantly shorter OS compared to those without alterations (5-year OS, 26.9% vs. 89.5%; hazard ratio [HR], 14.65; 95% confidential interval [CI], 2.995–71.68; *P* = 0.0009) (Fig. 2B). Similarly, patients with gains or losses of chromosome 7 displayed a significantly shorter OS compared to those without alterations (median OS, 14.6 months vs. not-reached; HR, 55.08; 95%CI, 5.983–507.1; *P* = 0.0004) (Fig. 2C). Conversely, no significant differences were observed when divided patients based on the presence of gains or losses in chromosome 3 (5-year OS, 66.3% vs. 80.1%; HR, 2.432; 95%CI, 0.4763–12.42; *P* = 0.2853), chromosome 5 (median OS, 26.97 months vs. not-reached; HR, 3.007; 95%CI, 0.3807–23.75; *P* = 0.2964), chromosome 9 (5-year OS, 58.3% vs. 80.7%; HR, 1.315; 95%CI, 0.3238–5.338; *P* = 0.7019), chromosome 12 (5-year OS, 66.7% vs. 76.8%; HR, 1.049; 95%CI, 0.1274–8.647; *P* = 0.9643), and chromosome 18 (5-year OS, 25% vs. 82.8%; HR, 4.665; 95%CI, 0.7273–29.93; *P* = 0.1043).

### NIPT-based liquid biopsy identifies MM and CLL patients with poorer prognosis

Next, to investigate disease-specific chromosomal alteration signature and clinical impact of these abnormalities, patients were divided based on diagnosis, and clinical characteristics, chromosomal abnormalities, and outcomes were compared. A total of 20 patients with plasma cell dyscrasias were included in this study with a median age of 57 years old (range, 49–64 years old) and a prevalence of females (65%). Diagnoses were: MM (*N* = 12, 60%); smoldering multiple myeloma (SMM; *N* = 5, 25%); plasma cell leukemia (PCL; *N* = 2, 10%); and systemic light chain amyloidosis (AL; *N* = 1, 5%) (Table 1). Monoclonal proteins (M-proteins) were IgG, IgA, and free light chain only (micromolecular MM) in 70%, 20%, and 10% of cases, respectively, with κ light chain represented in 65% of individuals. High-risk genetic abnormalities, including del(13q) or del(17p) detected by fluorescence in situ hybridization (FISH) or somatic mutations in *TP53* by next-generation sequencing, were detected in 20% of cases. Of note, karyotype analysis did not identify qualitative and/or quantitative alterations in any sample. Conversely, chromosomal abnormalities detected by NIPT were described in 25% of patients, with multiple abnormalities (≥ 3) in 20% of cases. When considering only patients with very high tumor burden (*N* = 6), including those with extramedullary disease or PCL, the rate of positive liquid biopsy was extremely high (83%; *P* = 0.0004). Moreover, patients with extramedullary diseases (both plasma cell dyscrasias or B-cell lymphomas) with positive liquid biopsy tended to have a shorter overall survival (OS) compared to those subjects with extramedullary disease without chromosomal abnormalities by NIPT or to those without extramedullary disease and with positive liquid biopsy (5-year OS, 51.5% vs. 94.4% vs 85.7%, with extramedullary disease and positive liquid biopsy vs without extramedullary disease and with positive liquid biopsy vs with extramedullary disease and positive liquid biopsy; *P* = 0.1939).

**Table 1:** Clinical features of patients with plasma cell dyscrasia

Characteristics	*N* = 20
Median age, years (range)	57 (49–64)
*Gender, n (%)*	
Male	8 (35)
Female	13 (65)
*Diagnosis, n (%)*	
MM	12 (60)
SMM	5 (25)
PCL	2 (10)
AL amyloidosis	1 (5)
*M-protein type, n (%)*	
IgG	14 (70)
IgA	4 (20)
Micromolecular	2 (10)
*Light chain type, n (%)*	
*κ*	13 (65)
*λ*	7 (25)
M-protein levels, g/dL, median (range)	2.5 (1.8–3.2)
High genetic risk abnormalities, *n* (%)	4 (20)
EMD, *n* (%)	6 (30)
Positive liquid biopsy, *n* (%)	5 (25)
3 ≥ genetic abnormalities	4 (20)
Positive liquid biopsy in PCL or EMD, n (%)	5 (83)
Alive, n (%)	15 (75)

*MM* multiple myeloma, *SMM* smoldering MM, *PCL* plasma cell leukemia, *AL* light chain, *Ig* immunoglobulin, *EMD* extramedullary disease

**Fig. 3:**
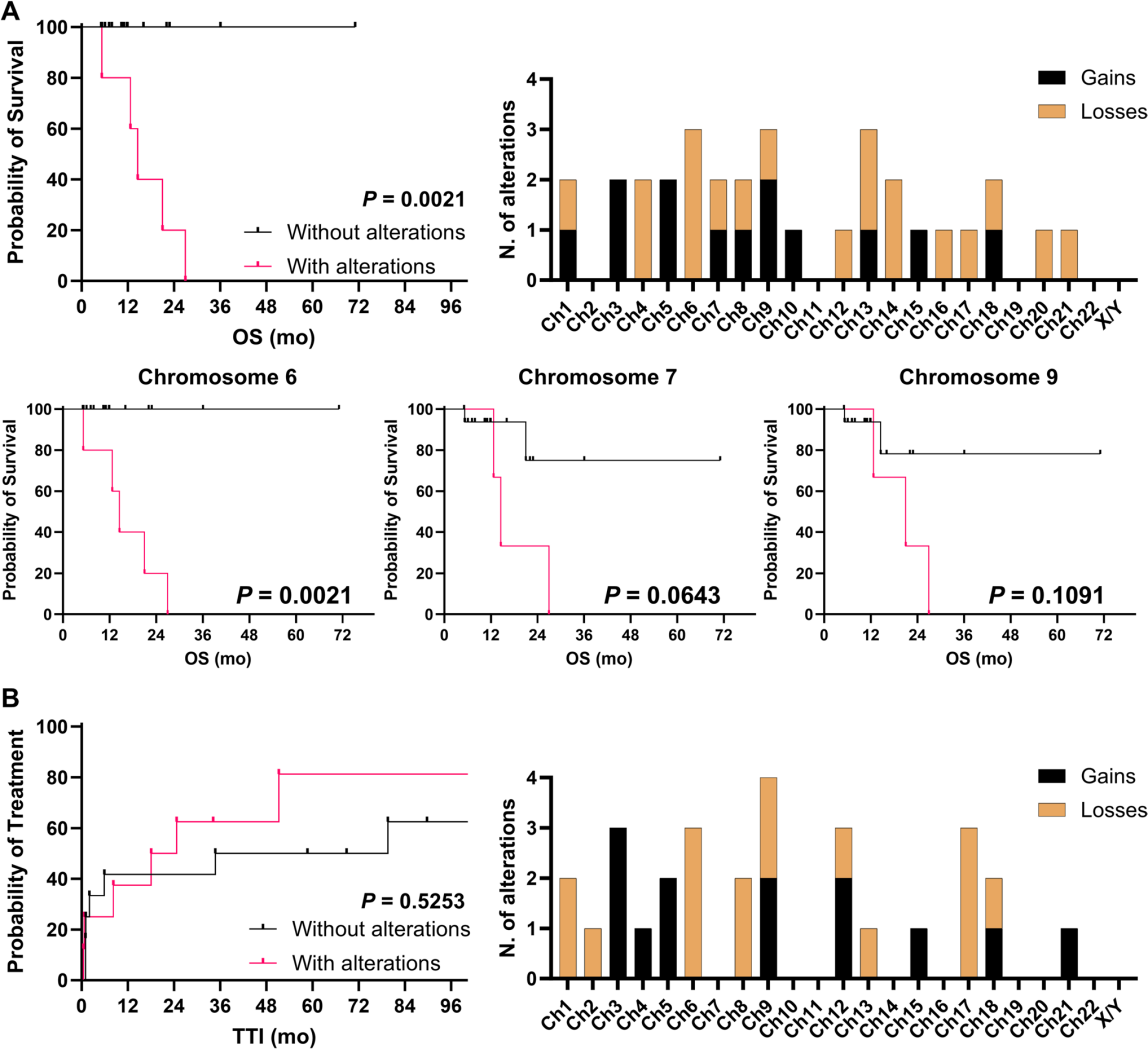
Clinical outcomes and frequency distribution in plasma cell dyscrasias and chronic lymphocytic leukemia (CLL). (**A**) Kaplan–Meier curves for overall survival (OS) of plasma cell dyscrasia patients with and without chromosomal abnormalities by NIPT, and by chromosome involved (chromosome 6, 7, and 9), and frequency distribution of alterations by category (gains or losses). (**B**) Time-to-treatment initiation (TTI) of CLL patients with and without chromosomal abnormalities by NIPT, and frequency distribution of alterations by category (gains or losses)

At the time of data cut, five subjects died (25%), and all of them had positive liquid biopsy at diagnosis, with a significantly shorter OS (median OS, not-reached *vs* 18 months, patients with genetic abnormalities detected by NIPT *vs* patients without abnormalities; HR, 80; 95%CI, 0.5–50; *P* = 0.002) (Fig. 3A). Among chromosomal abnormalities, losses of chromosome 6 (15% vs. 0%, losses vs. gains) and chromosome 9 (10% vs. 5%, losses vs gains) and gains of chromosome 13 (5% vs. 10%, losses vs gains) were more frequently observed (Fig. 3A). In particular, patients with chromosome 6 abnormalities showed a shorter OS compared to those without alterations by NIPT (median OS, 14.57 months vs not-reached, respectively; *P* = 0.0021), as well as for chromosome 7 (*P* = 0.0643) and chromosome 9 abnormalities (*P* = 0.1091) (Fig. 3A).

A total of 22 CLL patients were included with a median age of 66 years old (range, 62–64 years old) and a prevalence of males (64%) (Table 2). *TP53* mutations or del(17p) were found in 13% of cases by FISH or next-generation sequencing. Conversely, NIPT-based liquid biopsy was positive in 41% of patients, with 67% of them had ≥ 3 alterations. Nine patients (29%) died during follow-up period, and no differences were observed in OS between those subjects without chromosomal alterations detected by NIPT and those with abnormalities (5-years OS, 91.7% vs. 87.5%, without *vs* with abnormalities; HR, 0.7218; 95%CI, 0.08409 to 6.195; *P* = 0.7662). Conversely, time-to-treatment initiation (TTI) tended to be shorter in those CLL patients with chromosomal alterations by NIPT compared to those without abnormalities (median TTI, 21.4 months vs. 57.2 months, with vs without alterations; HR, 0.3741; 95%CI, 0.1298 to 1.078; *P* = 0.4703) (Fig. 3B). Among chromosomal abnormalities, losses of chromosome 6 (14% vs 0%, losses vs gains) and chromosome 17 (14% vs. 0%, losses vs gains) and gains of chromosome 3 (0% vs. 14%, losses vs gains) were more frequently observed (Fig. 3B).

**Table 2:** Clinical features of patients with chronic lymphocytic leukemia (CLL)

Characteristics	*N* = 22
Median age, years (range)	66 (62–64)
*Gender, n (%)*	
Male	14 (64)
Female	8 (36)
*RAI stage, n (%)*	
0–1	7 (32)
3-Feb	4 (18)
4	8 (36)
Not evaluable	3 (14)
del(17p)/*TP53* mutated, *n* (%)	3 (13)
Hemoglobin, g/dL, mean (range)	12.9 (8.3–15.7)
WBC, cells/µL, mean (range)	30,746 (3,010–183,650)
Platelets,/µL, mean (range)	145,161 (38,900–312,000)
β2-microglobulin, mg/dL, mean (range)	3.6 (0.2–14.6)
LDH, U/L, mean (range)	550.5 (170–2563)
Wait&watch, *n* (%)	6 (27)
Standard chemotherapy	12 (55)
Targeted therapy	2 (9)
Lost at follow-up	2 (9)
Positive liquid biopsy, *n* (%)	9 (41)
3 ≥ genetic abnormalities	6 (27)
Alive, n (%)	18 (82)

### Patients with aggressive B-cell lymphomas frequently have positive liquid biopsy

**Fig. 4:**
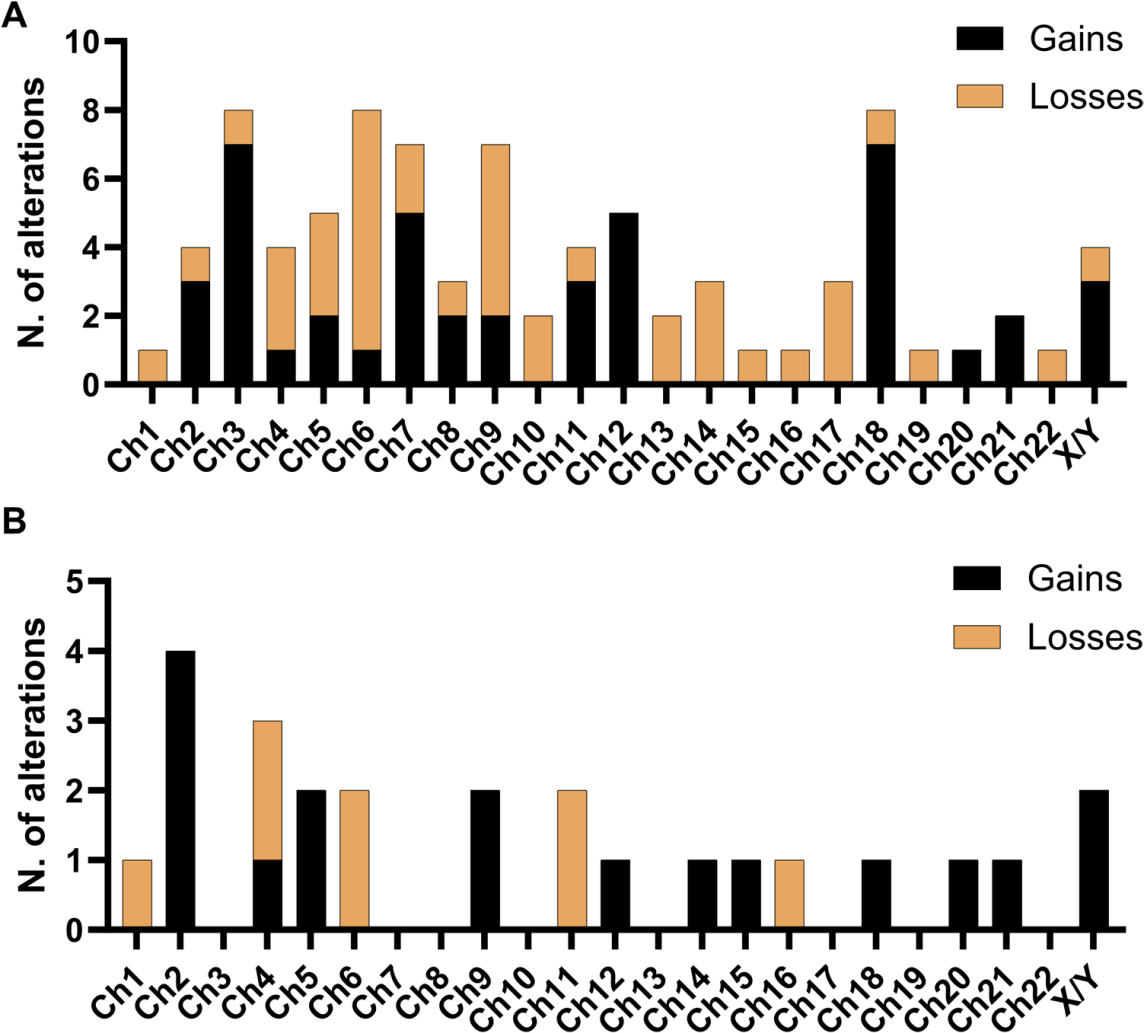
Frequency distribution of chromosomal abnormalities in lymphomas. Frequency distribution of alterations detected by NIPT by by category (gains or losses) in (**A**) B-cell lymphomas and (**B**) in Hodgkin lymphoma

In our B-cell lymphoma cohort (*N* = 26), patients were diagnosed with follicular lymphoma (FL; *N* = 6, 24%), marginal zone lymphoma (MZL; *N* = 3, 12%), mantle cell lymphoma (MCL; *N* = 3, 12%), and diffuse large B-cell lymphoma (DLBCL; *N* = 13, 52%) (Table 3). Median age was 68 years old (range, 62–76 years old), 52% of patients were males, and the majority had a late stage disease (80%). B-cell lymphoma patients had the highest rate of liquid biopsy positivity with 72% of cases showing chromosomal abnormalities and 61% of them with ≥ 3 alterations. In particular, patients with aggressive lymphomas had the highest percentage of positive liquid biopsy (85% for DLBCL and 67% for MCL), while indolent lymphomas the lowest (50% for FL and 33% for MZL), although not significant because of the small number of subjects in each subgroup (*P* = 0.12). Among chromosomal abnormalities, losses of chromosome 6 (28% vs. 4%, losses vs gains), chromosome 9 (20% vs. 8%, losses vs gains), and chromosomes 5 (12% vs. 8%, losses vs gains), and gains of chromosome 3 (4% vs. 28%, losses vs gains), chromosome 18 (4% vs. 28%, losses vs gains), chromosome 7 (8% vs. 20%, losses vs gains) and chromosome 12 (0% vs. 20%, losses vs gains) were more frequently observed over losses of chromosome regions (Fig. 4A). No differences in survival were observed based on NIPT positivity, because only one case of refractory disease and two deaths were registered, while 19 patients (77%) achieved a complete response with an OS rate of 92%.

**Table 3:** Clinical features of patients with B-cell lymphomas

Characteristics	*N* = 26
Median age, years (range)	68 (62–76)
*Gender, n (%)*	
Male	14 (54)
Female	12 (46)
*Diagnosis, n (%)*	
DLBCL	13 (50)
FL	6 (23)
MZL	3 (12)
MCL	3 (12)
B-cell lymphoma not otherwise specified	1 (3)
Clinical course, *n* (%)	
Indolent lymphoma	9 (36)
Aggressive lymphoma	16 (64).
*Stage, n (%) *	
I–II	5 (20)
III–IV	20 (80)
*IPI score$*	
Low	2 (15)
Low-intermediate	4 (31)
High-intermediate	5 (39)
High	2 (15)
*FLIPI score& *	
Low	1 (17)
Intermediate	1 (17)
High	4 (66)
*First-line treatments*	
R-COMP/CHOP/MACOP	11 (42)
G/R-Bendamustine	8 (31)
Rituximab	6 (23)
Cyclophosphamide + dexamethasone	1 (4)
Response to treatment, *n* (%)	
CR	19 (77)
RD	1 (4)
Not available	5 (19)
Positive liquid biopsy, n (%)	18 (72)
3 ≥ genetic abnormalities	11 (44)
Alive, *n* (%)	23 (92)

*DLBCL* diffuse large B-cell lymphoma, *FL* follicular lymphoma, *MZL* marginal zone lymphoma, *MCL* mantle cell lymphoma, *IPI* International Prognostic Index, *FLIPI* Follicular Lymphoma International Prognostic Index, *CR* complete remission, *RD*, refractory disease, ^$^ percentage on *DLBCL* patients, ^&^ percentage on FL patients

A total of 10 HL patients were included with a median age of 26 years old (range, 22–54 years old) and females were 50% of them. Nodular sclerosis was the most common histotype (90%), and the majority of patients had an early stage disease (*N* = 7; 70%) (Table 4). Liquid biopsy was positive in 50% of cases, and 80% of them displayed ≥ 3 abnormalities. Among chromosomal abnormalities, gains of chromosome 2 (40% vs. 0%, gains vs losses), chromosome 5 (20% vs. 0%, gains vs losses), and sex chromosomes (20% vs. 0%, gains vs losses), and losses of chromosome 4 (20% vs. 10%, losses vs gain) and chromosomes 6 and 11 (20% vs. 0%, each, losses vs gain) were more frequently described over losses of chromosome regions (Fig. 4B). First-line treatment consisted of dose-dense or standard ABVD (90% or 10%, respectively), and complete remission was observed in 70% of cases; while two cases had primary refractory disease, and one of them had positive liquid biopsy. At data cut, OS was 90% with one death occurred during induction therapy.

**Table 4:** Clinical features of patients with Hodgkin lymphomas

Characteristics	*N* = 10
Median age, years (range)	26 (22–54)
*Gender, n (%)*	
Male	5 (50)
Female	5 (50)
*Histological variant, n (%)*	
Nodular sclerosis	9 (90)
Lymphocyte-predominant	1 (10)
*Stage, n (%)*	
I-II	7 (70)
III-IV	3 (30)
Treatment, *n* (%)	
Dose-dense ABVD	9 (90)
ABVD	1 (10)
*Response to treatment, n (%)*	
CR	7 (70)
RD	2 (20)
Death during induction	1 (10)
Positive liquid biopsy, n (%)	5 (50)
3 ≥ genetic abnormalities	4 (40)
Alive, n (%)	9 (90)

*ABVD* doxorubicin (Adriamycin), bleomycin, vinblastine, dacarbazine, *CR* complete remission, *RD* refractory disease

## Discussion

Cytogenetics analysis is essential for risk stratification of hematological malignancies, including acute leukemias, lymphomas, and plasma cell dyscrasias [26]. Certain chromosomal abnormalities are recurrent and specific for disease, such as the Philadelphia chromosome—t(9;22) (q34;q11)—in chronic myeloid leukemia or B-cell acute lymphoblastic leukemia, while several clinical entities lack of genetic and chromosomal signatures thus of potential pharmacological targets [27, 28]. Moreover, accurate MRD monitoring, an important prognostic marker, is challenging, because it relies on sensibility and specificity of used method and disease burden [29, 30]. Liquid biopsy from cfDNA is a sensitive method for tumor genotyping, prognostic definition, and disease monitoring, and its utility is increasingly studying in hematological malignancies [4]. Here, we investigated clinical utility of NIPT-based liquid biopsy in plasma cell dyscrasias, B-cell lymphomas for identification of chromosomal abnormalities and correlation with clinical outcomes, showing a comparable sensitivity of NIPT to standard routinary used methods, such as FISH and karyotyping. Moreover, the presence of chromosomal abnormalities in cfDNA detected by NIPT was associated with worse outcomes in plasma cell dyscrasia and CLL, while B-cell lymphomas displayed a greater genomic heterogeneity.

Liquid biopsy can be employed for detection of tumor-related somatic mutations in cfDNA, based on the evidence that highly proliferating tumor cells directly release genomic materials or indirectly through apoptotic bodies and debris [31]. Plasma cfDNA is predominantly of hematopoietic origin with approximately 55% from white blood cells and 30% from erythrocyte progenitors [32]. In solid tumors or hematological malignancies, cfDNA comprises of nucleic acids derived from both hematopoietic and tumor cells, known as circulating tumor DNA (ctDNA), and its levels vary across clinical entities, disease stage, and tumor burden [4]. Therefore, cfDNA can be used as a surrogate sample for detection of tumor-related genomic alterations, and quantification of circulating mutation frequency highly mirrors disease burden [33]. Cancer personalized profiling by deep sequencing (CAPP-Seq) and PhasED-seq is highly accurate technologies for identification of somatic mutations and for disease monitoring in DLBCL and HL; however, these methodologies are time consuming, expensive, require high specialized personnel, and are low reproducible across laboratories [4]. Moreover, data normalization, assay standardization, and validation of liquid biopsy methodologies are still challenging, because of several technical issues, including choice of housekeeping genes, normalization methods, nucleic acid degradation rate, or small amounts of ctDNA, especially in those subjects with low tumor burden [12]. Other methodologies, such as droplet digital polymerase chain reaction (ddPCR) or beads, emulsion, amplification, and magnetics (BEAMing), can be also used for liquid biopsy analysis, as ddPCR can detect rare mutations in low genomic material (as low as 0.01–1.0%) [34, 35]. Whole exome or genome sequencing evaluate all mutations or the entire tumor genome with the ability to identify deleterious alterations, variants of unknown significance, or novel variants; however, they are not routinarly used in clinical practice [36]. NIPT is a methodology employed for minimally invasive analysis of fetal DNA in maternal circulation with the purpose to identify fetal chromosomal abnormalities before birth, by avoiding invasive diagnostic tests [37]. Recently, it has been described that positive NIPT in the absence of fetal chromosomal alterations confirmed by amniocentesis or chorionic villus sampling can be an alert for maternal unknown silent cancers [23]. Therefore, in this study, we tested feasibility and clinical utility of NIPT-based liquid biopsy in several hematological cancers, including plasma cell dyscrasia, HL, and B-cell lymphomas. In our cohort, half of patients had positive NIPT with a median of three alterations, more frequently of chromosomes 6, 9, 3, 18, 5, 2, 4, 7, and 12. Of those subjects with positive test, the majority had B-cell lymphomas (49%), while the remaining received a diagnosis of CLL (24%), MM or HL (13.5% each). Interestingly, MM patients showed chromosomal alterations by FISH or carried *TP53* mutation by NGS only in 20%, while conventional karyotyping without plasma cell enrichment was negative in all cases. Indeed, conventional cytogenetics analysis assesses the presence of chromosomal abnormalities in metaphases of proliferating cells under standard culture conditions, while proliferation rate of plasma cells is limited and requires certain stimuli in culture medium, such as lipopolysaccharide, various cytokines (e.g., interleukin[IL]-6), or synthetic DSP30, an oligonucleotides containing CpG motif [38]. However, conventional karyotyping with plasma cell enrichment is not always feasible in routinely clinical practice and fails to recognize cryptic translocations of the IgH locus or 17p deletions [39]. Here, we documented a comparable sensitivity of NIPT to other methodologies in detecting chromosomal alterations in MM, even using peripheral blood specimens and without plasma cell enrichment procedures, especially in those subjects with extramedullary disease. Concordance of results was observed in 11 cases (55%) and discordance in 9 (45%), of whom only two displayed abnormalities by karyotyping and not by NIPT, probably because of balanced alterations (a t(11;14) and a del17p + trisomy 17), while four had positive NIPT and negative or failed FISH/karyotyping. Similarly, genetic alterations were found in 13% of CLL by FISH or NGS, while chromosomal abnormalities were described in 41% of them by NIPT. In particular, concordance of results was described in six cases (27%), while discordance in the remaining 16 patients (73%), of whom six displayed abnormalities by FISH/karyotyping while not by NIPT, and nine had positive NIPT with negative or failed conventional tests, confirming the potential clinical utility of NIPT-based liquid biopsy compared to standard analyses. In B-cell lymphomas, conventional karyotyping is not routinely performed, as disease-specific rearrangements or mutations are directly detected on histological samples for diagnosis confirmation [22]. In our study, B-cell lymphomas had the highest NIPT positivity, especially those subjects with aggressive lymphomas (DLBCL and MCL).

In MM, del(17q13), translocations involving IgH loci on chromosome 14, and chromosome 17 and 1 alterations are included in current risk stratification systems [19]. In our study, abnormalities were frequently described in chromosomes 6, 9, 3, 18, 5, 2, 4, 7, and 12, with losses of chromosomes 6 and gains of chromosome 7 negatively impacting on clinical outcomes regardless underlying diseases. In particular, losses of chromosome 6 were commonly found in MM, B-cell lymphomas, HL, and CLL, while losses of chromosome 9 were found in MM and B-cell lymphomas, as well as gains of chromosome 3 in CLL and B-cell lymphomas. Some abnormalities were disease-specific, such as gains of chromosome 13 in MM, losses of chromosome 17 in CLL, losses of chromosome 5 and gains of chromosomes 7, 12, and 18 in B-cell lymphomas. Surprisingly, NSHL showed a completely different signature, except for losses of chromosome 6, compared to other studied hematological malignancies, as patients displayed gains of chromosomes 2 and 5 and of sex chromosomes, and losses of chromosomes 4 and 11. Whole chromosome 6 loss determines lost of heterozygosity (LOH) of two important genomic regions located on 6p, the major histocompatibility complex region on 6p21.31, and on 6q, a tumor-suppressor gene region [40, 41]. In cancers, 6p copy number neutral (CN) LOH is a very common mechanism of immune surveillance escaping, as lost of 6p arm determines the lack of expression of HLA class I alleles on cell surface, thus favoring the escape of cells from autologous attack of cytotoxic T cells [40–43]. Hodgkin and Reed-Stenberg cells in HL can also frequently use this mechanism to evade immune surveillance [44]. In our study, using NIPT-based technologies for liquid biopsy analysis, we demonstrated that whole chromosome 6 loss or 6p LOH are common alterations in hematological malignancies, not only in those with an immune-mediated pathogenesis, such as acquired bone marrow failure syndromes and HL. Moreover, losses of the long arm of chromosome 6 are frequently in solid tumors, including melanoma, renal cell carcinoma, salivary gland adenocarcinoma, or ovarian carcinoma, and in hematologic malignancies, such as acute lymphoblastic leukemia and B-cell lymphomas, occurring in at least 32 and 7% of patients, respectively [44]. Among B-cell lymphomas, deletions of 6q are commonly observed in CLL, MM, DLBCL, and FL, in which are associated with adverse clinical outcomes [41]. Here, we showed that chromosome 6 losses were frequent abnormalities in hematological malignancies and were associated with a worse prognosis, regardless underlying diseases, as well as for chromosome 7 gains. Usually, in hematological malignancies, including myelodysplasia, acute myeloid leukemia, and bone marrow failure syndromes, monosomy 7 or del(7q) are the most frequent alteration of chromosome 7 and are associated with poor clinical outcomes [45]. Conversely, gains of chromosome 7 have been proposed as the evolutionary first driver event in glioblastomas through dysregulation of stemness-related gene cluster, the HOX genes, expression. In early stage, gene copy gains loci are hypermethylated in a compensation mechanism, while in late stage, cells escape from hypermethylation and HOX-related genes are upregulated conferring stemness properties and drug resistance [46]. In our study, gains of chromosome 7 might be frequent also in hematological malignancies, especially in aggressive diseases, such as MM with extramedullary involvement and DLBCL, and could be associated with a worse prognosis.

Our research has some limitations: (i) the small number of patients in each hematological disease group (e.g., B-cell lymphoma subgroups) was limited, as we reported a single-center real-life study enrolling consecutive patients over a short period of time; (ii) lack of cytogenetics data in lymphomas and of plasma cell enriched karyotyping in MM; and (iii) a short period of observation at data cut, especially for lymphoma patients because of the prospective nature of our investigation.

In conclusions, liquid biopsy is increasingly used as a surrogate diagnostic minimally invasive tool for detection of known somatic mutations in solid tumors and hematological malignancies. Current methods for liquid biopsy have several limitations, making its routinely use in clinical practice difficult. Here, we showed utility of NIPT-based liquid biopsy in hematological malignancies, with a better sensitivity in identifying chromosomal abnormalities in circulating cfDNA compared to standard cytogenetics analysis, such as FISH and conventional karyotyping, that use cultured cells from tumor tissues (e.g., the bone marrow). Moreover, screening for chromosomal abnormalities in the whole genome displayed recurrent alterations across hematological malignancies with worse clinical outcomes, and potential disease-specific chromosomal signature. However, prospective studies on larger cohorts are needed to validate our data and clinical utility of NIPT-based liquid biopsy in routinely clinical practice.

## Data Availability

Data are available upon request by the authors. The authors declare that the material is original, has not been published before nor is under consideration in any journal.
